# Deletion of miR‐33, a regulator of the ABCA1–APOE pathway, ameliorates neuropathological phenotypes in APP/PS1 mice

**DOI:** 10.1002/alz.14243

**Published:** 2024-09-30

**Authors:** Mason Tate, H. R. Sagara Wijeratne, Byungwook Kim, Stéphanie Philtjens, Yanwen You, Do‐Hun Lee, Daniela A. Gutierrez, Daniel Sharify, Megan Wells, Magdalena Perez‐Cardelo, Emma H. Doud, Carlos Fernandez‐Hernando, Cristian Lasagna‐Reeves, Amber L. Mosley, Jungsu Kim

**Affiliations:** ^1^ Stark Neuroscience Research Institute Indiana University School of Medicine Indianapolis Indiana USA; ^2^ Medical Neuroscience Graduate Program Indiana University School of Medicine Indianapolis Indiana USA; ^3^ Department of Biochemistry and Molecular Biology Indiana University School of Medicine Indianapolis Indiana USA; ^4^ Department of Medical & Molecular Genetics Indiana University School of Medicine Indianapolis Indiana USA; ^5^ Vascular Biology and Therapeutics Program Yale University School of Medicine New Haven Connecticut USA; ^6^ Department of Comparative Medicine Yale University School of Medicine New Haven Connecticut USA; ^7^ Yale Center for Molecular and System Metabolism, Yale University School of Medicine New Haven Connecticut USA; ^8^ Center for Proteome Analysis Indiana University School of Medicine Indianapolis Indiana USA

**Keywords:** ABCA1, Alzheimer's disease, amyloid, apolipoprotein E, lipid metabolism, microRNA‐33

## Abstract

**INTRODUCTION:**

Rare variants in *ABCA1* increase the risk of developing Alzheimer's disease (AD). ABCA1 facilitates the lipidation of apolipoprotein E (apoE). This study investigated whether microRNA‐33 (miR‐33)‐mediated regulation of this ABCA1–APOE pathway affects phenotypes of an amyloid mouse model.

**METHODS:**

We generated *mir‐33^+/+^*;*APP/PS1* and *mir‐33^−/−^*;*APP/PS1* mice to determine changes in amyloid pathology using biochemical and histological analyses. We used RNA sequencing and mass spectrometry to identify the transcriptomic and proteomic changes between our genotypes. We also performed mechanistic experiments by determining the role of miR‐33 in microglial migration and amyloid beta (Aβ) phagocytosis.

**RESULTS:**

*Mir‐33* deletion increases ABCA1 levels and reduces Aβ accumulation and glial activation. Multi‐omics studies suggested miR‐33 regulates the activation and migration of microglia. We confirm that the inhibition of miR‐33 significantly increases microglial migration and Aβ phagocytosis.

**DISCUSSION:**

These results suggest that miR‐33 might be a potential drug target by modulating ABCA1 level, apoE lipidation, Aβ level, and microglial function.

**Highlights:**

- Loss of microRNA‐33 (miR‐33) increased ABCA1 protein levels and the lipidation of apolipoprotein E.
- Loss of miR‐33 reduced amyloid beta (Aβ) levels, plaque deposition, and gliosis.
- mRNAs and proteins dysregulated by miR‐33 loss relate to microglia and Alzheimer's disease.
- Inhibition of miR‐33 increased microglial migration and Aβ phagocytosis in vitro.

## 1 BACKGROUND

Alzheimer's disease (AD) is pathologically characterized by the aggregation of amyloid beta (Aβ) peptides and tau proteins along with extensive neuroinflammation.^1^ A small subset of AD cases is familial and caused by mutations in genes involved in Aβ peptide generation. The majority of cases are sporadic, with no known familial AD genetic mutations. For these cases, the strongest genetic risk factor is the apolipoprotein E (*ΑPOE*) ε4 allele.^2^, ^3^ ApoE is the major cholesterol transport protein in the central nervous system.^4^ Importantly, ATP binding cassette subfamily A member 1 (*ABCA1*) was recently identified as a novel risk factor gene for developing AD.^5^, ^6^ ABCA1 mediates the efflux of cholesterol and phospholipids onto lipid‐poor apoE in the brain. Mice lacking *Abca1* have altered lipid metabolism, most notably poorly lipidated and abnormally small apoE lipid particles.^7^ These small, poorly lipidated apoE particles are similar in size to apoE ε4 particles, further highlighting apoE lipidation as a crucial factor in AD pathogenesis.^8^, ^9^

Amyloidosis mouse models have been valuable tools for studying the pathogenesis of AD.^10^ These mouse models recapitulate many of the amyloid pathological features present in AD patients, including the generation of Aβ peptides, plaque deposition, and neuroinflammation. Interestingly, several studies have shown that knocking out *Abca1* in amyloidosis mouse models increases the formation of Aβ peptides and amyloid plaque deposition.^11^, ^12^ Conversely, increasing *Abca1* expression, and subsequently apoE lipidation, reduces Aβ peptides and plaque deposition.^13^ Thus, it is a promising approach to identify therapeutic targets to increase *ABCA1* expression and reduce amyloid pathology.

Mature microRNAs (miRs) are a class of small, non‐coding RNAs that bind to the 3′unstranslated region of their target messenger RNAs (mRNAs), leading to inhibition and/or degradation of the mRNA.^14^ Previously, it was shown that miR‐33 negatively regulates ABCA1 in both the periphery and the brain.^15^, ^16^, ^17^, ^18^ Furthermore, we demonstrated that deletion of the *mir‐33* gene increases the lipidation of apoE in the brains of wild‐type mice.^17^ However, the functional effect of *mir‐33* deletion in the context of amyloid pathology has not been investigated. Given that *APOE* and *ABCA1* are known genetic risk factors for AD, and miR‐33 affects the ABCA1–APOE pathway, understanding the role of miR‐33 in the pathogenesis of AD is important. In this study, we aimed to address this critical gap in knowledge by generating *mir‐33^−/−^* mice crossed with an amyloidosis transgenic mouse model. We demonstrated that deletion of *mir‐33* significantly increased ABCA1 protein levels, increased the lipidation of apoE, and reduced amyloid pathology and gliosis at 8 months of age. To identify the underlying mechanisms by which the deletion of *mir‐33* causes these beneficial effects, we used a multi‐omics approach by performing RNA sequencing (RNA‐seq) and mass spectrometry–based proteomics. We found that the loss of *mir‐33* regulates pathways relating to microglial activation and microglial migration. Most importantly, we validated the prediction based on the multi‐omics data by performing mechanistic cell biology experiments. We found that inhibition of miR‐33 increases the migration of microglia. Additionally, inhibition of miR‐33 increases the microglial phagocytosis of Αβ aggregates. Taken together, our data suggest that miR‐33 may be a potential target for ameliorating amyloid pathology and dysfunctional glial phenotypes.

## 2 METHODS

### 2.1 Mice

All animals used in this project followed the guidelines of the institutional animal care and use committee at the Stark Neurosciences Research Institute and Indiana University School of Medicine. We generated our mouse model by crossing *mir‐33^+/+^* or *mir‐33^−/−^* mice^19^ with *APPsw/PSEN1Δ9* (*APP/PS1*) transgenic mice overexpressing Swedish mutant *APP695* and *PSEN1* with an exon 9 deletion.^20^ This *APP/PS1* mouse model exhibits sex differences in amyloid pathology.^21^ We used female mice for this study due to their significantly higher levels of amyloid deposition compared to males. Mice were sacrificed at 8 months of age.

### 2.2 Brain sample collection and sample processing

Mice were anesthetized with 250 mg/kg tribromoethanol by intraperitoneal injection and transcardially perfused with ice‐cold phosphate‐buffered saline (PBS). For each mouse, one hemibrain was fixed in 4% paraformaldehyde for 24 hours at 4°C followed by storage in 30% sucrose. The other hemibrain was dissected and samples were stored at −80°C . PBS‐, radioimmunoprecipitation assay (RIPA)‐, and guanidine‐soluble proteins were sequentially extracted from brain tissues as previously described.^22^

### 2.3 Meso Scale Discovery Aβ electrochemiluminescence‐based enzyme‐linked immunosorbent assay

The concentrations of Aβ peptides in the hippocampus and cortex from the guanidine fraction were determined using the V‐PLEX Plus Aβ Peptide Panel 1 (6E10) Kit (Meso Scale Discovery [MSD]; K15200E) per manufacturer's instructions. Signals were measured on a MESO QuickPlex SQ 120 multiplexing imager.

### 2.4 Histological analysis

Fixed hemibrains were frozen and serial coronal sections (20 µm thick) were obtained from rostral (bregma −1.22 mm) to caudal (bregma −2.70 mm) regions using a cryostat (Leica Biosystems; CM 1860). The sections were stored in a cryoprotectant solution (50% glycerol in 0.1 M PBS) at −20°C. Images were quantified using Fiji (ImageJ; v1.8.0, NIH)^23^ and CellProfiler (v4.2.1).^24^ Three sections from each mouse spaced 460 µm apart were used for staining and the averages were used for quantification.

**RESEARCH IN CONTEXT**

1. **Systematic review**: The authors reviewed the literature on the regulation of *ABCA1* in the context of Alzheimer's disease (AD) through a PubMed search. Recent studies have identified rare variants in the *ABCA1* gene that are associated with an increased risk of developing AD. Considering *ABCA1*’s role in lipid metabolism and its interaction with apolipoprotein E (*APOE*), identifying upstream regulators of *ABCA1* could hold therapeutic potential for AD. However, it remains unknown whether non‐coding RNAs can regulate the ABCA1–APOE pathway, thereby influencing AD pathology.
2. **Interpretation**: Deletion of microRNA‐33 (mir‐33) increases ABCA1 levels, leading to an increase in apoE lipidation. In an amyloidosis mouse model, miR‐33 deletion reduces the levels of amyloid beta, accumulation of amyloid plaques, and neuroinflammation. These beneficial effects are mediated through different downstream mechanisms in astrocytes and microglia.
3. **Future directions**: Future studies are warranted to investigate the cell type–specific role of miR‐33 in the brain using new mouse models and to determine the therapeutic potential of miR‐33 inhibitors in AD.

#### 2.4.1 Immunohistochemistry

To determine Aβ plaque deposition, the sections were probed with an Aβ‐specific 82E1 antibody (1:500, IBL‐AMERICA; IBL10323). The sections were treated with 10% methanol and 3% hydrogen peroxide in PBS for 10 minutes. The sections were blocked with 4% skim milk in PBS solution for 1 hour at room temperature, then incubated with mouse anti‐82E1 with 0.25% Triton X‐100 in 2% skim milk PBS solution at 4°C overnight. The sections were then incubated with a biotinylated anti‐mouse antibody (Vector Laboratories; BA‐9200) in a blocking solution for 1 hour at room temperature. Antibody binding was detected with Vectastain ABC Elite (Vector Laboratories; PD6101) and DAB development kits (Vector Laboratories; SK‐4100) according to the manufacturer's instructions. To determine glial activation, the sections were probed with an anti‐CD45 antibody (1:500, Bio‐Rad; MCA1388) following the same protocol described above for anti‐82E1 antibody staining. To determine the accumulation of mature fibrillar plaques, the sections were stained with X‐34. Sections were permeabilized with 0.25% Triton X‐100 in PBS and stained with 10 µM X‐34 solution consisting of 40% ethanol and 0.02 N sodium hydroxide in PBS for 30 minutes at room temperature. For immunofluorescence staining, the sections were blocked with 5% normal donkey serum (NDS) in PBS for 1 hour at room temperature. The sections were incubated with primary antibodies in 2.5% NDS at 4°C overnight with anti‐ionized calcium binding adaptor molecule 1 (IBA1; 1:1000, Abcam; ab178846) and anti‐glial fibrillary acidic protein (GFAP; 1:1000, Dako Ommis; Z0334) antibodies. The sections were then incubated with Alexa Fluor 488 and/or Alexa Fluor 568 fluorescent antibodies.

#### 2.4.2 Image acquisition

Images were taken on a Leica DM6 B inverted microscope at 10x magnification. Three sections per mouse were analyzed and averaged. For each section, the entire hippocampal area was segmented for analysis. The cortical area was segmented to include the entire cortex from the retrosplenial area moving laterally to the somatosensory cortex. Image analysis was performed in ImageJ (v1.8.0) and CellProfiler (v4.2.1).

### 2.5 Western blot analysis

Denatured western blot samples were prepared from the PBS and RIPA lysates and performed as previously described.^25^ Native condition western blot samples were prepared from the PBS lysate in Native Sample Buffer (Bio‐Rad; 1610738). A total of 15 µg of protein were loaded onto 4% to 20% gradient Criterion TGX gels (Bio‐Rad; 5671095), separated by gel electrophoresis (avoiding the use of any detergents and running at 4°C), and transferred to polyvinylidene difluoride membranes (Millipore; IPVH00010). The membranes were probed with the following primary antibodies: anti‐82E1 (1:500, IBL‐AMERICA; IBL10323), anti‐GAPDH (1:5000, Santa Cruz; sc‐25778), anti‐apoE (1:1000, ABclonal; A16344), anti‐ABCA1 (1:1000, Cell Signaling; 96292), and anti‐β‐Actin (1:50,000, Sigma; A1978). The chemiluminescent signal was determined with ECL (GE Healthcare) using the Amersham Imager 680 (GE Healthcare). Densitometry quantification was performed in the program ImageQuant TL v8.2.0.0 (GE Healthcare). We used the GAPDH or β‐Actin signal for the normalization of denatured western blots. Quantification of the high molecular weight native apoE western blot data was normalized to the level of total apoE in each sample.

### 2.6 Mouse apoE enzyme‐linked immunosorbent assay

The concentrations of apoE protein levels from hippocampal PBS lysate were determined using the Mouse Apolipoprotein E enzyme‐linked immunosorbent assay (ELISA) Kit (Abcam; ab215086) per the manufacturer's instructions.

### 2.7 ApoE immunoprecipitation and cholesterol analysis

ApoE protein was immunoprecipitated using the Dynabeads Protein G Immunoprecipitation Kit (Thermo Fisher Scientific; 10007D). Anti‐apoE antibody (HJ6.3) was conjugated to the Dynabeads to immunoprecipitate apoE protein from cortical PBS lysate.^26^ Samples were incubated with the antibody‐conjugated Dynabeads for 30 minutes. The Dynabeads were washed with PBS three times, and then the immunoprecipitated apoE (IP) was eluted from the Dynabeads with 0.1% Triton‐X 100 in PBS. We performed denatured western blot analysis with the anti‐apoE antibody (1:1000, ABclonal; A16344) to determine levels of APOE from the IP samples.

Cholesterol levels from the IP samples were measured with the Amplex Red Cholesterol Assay Kit (Thermo Fisher Scientific; A12216) per the manufacturer's instructions. The concentration of cholesterol from the IP samples were normalized with the amount of apoE protein determined from western blot analysis from each IP sample.

### 2.8 Quantiative polymerase chain reaction (qPCR)

Total RNA was extracted as previously described.^25^ For the determination of mature miR, RNA was reverse transcribed to cDNA using the miScript II RT Kit (Qiagen, 218160) and quantitative polymerase chain reaction (qPCR) was performed with the miScript SYBR Green PCR Kit (Qiagen; 218073). For the determination of mRNA transcripts, RNA was reverse transcribed to cDNA using the High‐Capacity cDNA Reverse Transcription Kit (Thermo Fisher Scientific; 4368814) and qPCR performed with Fast SYBR Green (Thermo Fisher Scientific; 4385612). We performed qPCR using the QuantStudio 3 thermocycler (Applied Biosystems) 4385612. Primer information for miR‐33, U6, *Ldlr*, *Lrp1*, and *Gapdh* can be found in Table S1 in supporting information. We used endogenous U6 expression or *Gapdh* expression for normalization. Changes in expression were quantified as 2^−∆∆Ct^ in the program QuantStudio v1.5.0 (Applied Biosystems).

### 2.9 RNA‐seq

Our RNA‐seq experiment was outsourced to Lexogen Inc. (Austria) and conducted in the same way as previously described.^25^ RNAs isolated from cortical tissue were used for RNA‐seq. Differential gene expression analysis was performed on the resulting gene read count files using DESeq2 in R v.4.0.4^27^ using the DESeqDataSetFromHTSeqCount function with the design argument ∼ group (*APP/PS1*; *mir‐33* genotype). Genes with a total read count of ≤ 10 were removed from the dataset. Results were plotted using the ggplot2 package.^28^

### 2.10 Proteomics

#### 2.10.1 Peptide preparation

The protein lysates from the cortical RIPA fractions were used for proteomics. Proteins were precipitated using methanol/chloroform precipitation,^29^ sonicated, and clarified. A total of 75 µg of precipitated protein was similarly processed as previously described.^30^ The proteins were treated with tris(2‐carboxyethyl)phosphine followed by chloroacetamide. The samples were digested with Lys‐C (Promega), then PNGase F (New England BioLabs), then Trypsin Platinum (Promega), and quenched with trifluoroacetic acid.^31^, ^32^ The peptides were desalted, dried, and resuspended in triethylammonium bicarbonate. A total of 50 µg of peptides from each sample were labeled with TMTpro (Thermo Fisher Scientific)^31^ per manufacturer instructions. The labeling reaction was quenched with hydroxylamine, mixed, dried, desalted, resuspended with 0.1% formic acid (FA) and 70% acetonitrile (ACN), and lyophilized. Lyophilized peptides were fractionated using Thermo UltiMate 3000 high‐performance liquid chromatography (HPLC) with 10 mM ammonium formate pH 10 in water (buffer A) and 95% ACN (buffer B). Fractions were collected at 1 mL/minute for 110 minutes, interval concatenated into 48 fractions, dried, and resuspended in FA prior to liquid chromatography mass spectrometry (LC‐MS).^33^, ^34^, ^35^

#### 2.10.2 LC‐MS

Nano‐LC‐MS/MS analyses were performed on an EASY‐nLC HPLC system coupled to Orbitrap Eclipse Tribrid mass spectrometer with FAIMS Pro Interface (Thermo Fisher Scientific). Each fraction was loaded onto an Aurora Ultimate C18 column (IonOpticks). Peptides were eluted from 6% to 34% with buffer B (80% ACN with 0.1% FA). The mass spectrometer was operated in positive ion mode with a 4 second cycle time data‐dependent acquisition method with advanced peak determination and Easy‐IC (internal calibrant) with other mass spectrometry settings as previously described.^36^ The data were recorded using Thermo Fisher Scientific Xcalibur 4.3 software.

#### 2.10.3 Data analysis

The resulting RAW files were analyzed in Proteome Discover 2.5 using default settings unless specified with *Mus musculus* UniProt Reference Proteome FASTA (UP000000589, downloaded on 07/2021) plus common contaminants. SEQUEST‐HT searches were conducted with a maximum number of three missed cleavages, precursor mass tolerance of 10 ppm, and a fragment mass tolerance of 0.02 Da. Static modifications used for the search were carbamidomethylation on cysteine residues; TMTpro label on lysine residues and the N‐termini of peptides. Dynamic modifications used for the search were the TMTpro label on deamidation of asparagine; oxidation of methionine; phosphorylation on serine, threonine, or tyrosine; and protein N‐terminal modification with acetylation, methionine loss, or acetylation with methionine loss. Tandem mass tag (TMT) ion intensities were normalized by total peptide amount with no scaling and used lot‐specific TMTpro isotopic impurities. The genotype mean abundances and *t* test *p* values were calculated. Fold change for each protein is the mean abundance of *mir‐33* ^−/−^;*APP/PS1* samples divided by the mean abundance of *mir‐33* ^+/+^;*APP/PS1*.

### 2.11 Pathway and network analyses

Enrichment analyses were performed using MetaCore software (version 22.1; build 70800). The “Enrichment Analysis” function in MetaCore was used with both the RNA‐seq and proteomics data sets to determine Pathway Map enrichment. The “Compare Experiments” function in MetaCore was used with both the RNA‐seq and mass spectrometry data sets to determine Gene Ontology (GO) processes enrichment with the shared significant gene products. Any enrichment term with a ‐log(*p* value) ≥ 1.3 was considered significant.

### 2.12 miRNA target analysis

We used the R package multiMiR (v.1.20.0, Database Version: 2.3.0)^37^ to determine whether the deletion of mir‐33 increased the predicted or experimentally validated targets of miR‐33 targets. Proteins with a *p* value ≤ 0.05 and a fold change > 1 (increased abundance in the *mir‐33* ^−/−^;*APP/PS1* genotype) were searched across all miR databases available through multiMiR for targets of mmu‐miR‐33‐5p or mmu‐miR‐33‐3p. Selection required predictions from at least two miRNA databases.

### 2.13 In vitro cell culture

BV2 cells were plated in either 96‐well or 24‐well plates at densities of 2 × 10^4^ or 1 × 10^5^ cells/well, respectively, in Dulbecco's modified Eagle medium media containing 2% fetal bovine serum (Gibco; 10082‐147) and 1% penicillin/streptomycin (Gibco; 15140‐122). Cells were reverse transfected with the following constructs: miR‐33 mimic (Qiagen; GeneGlobe ID: GYM00473393‐ADA), miR Inhibitor control (Qiagen; GeneGlobe ID: YI00199006‐ADA), and miR‐33 Inhibitor (Qiagen; GeneGlobe ID: YI04102039‐ADA). Cells were transfected with Lipofectamine RNAiMAX (Invitrogen; 13778150) with a final siRNA concentration of 20 nM per construct for 24 hours, then the media was changed to fresh growth media. For in vitro experiments, “Ctrl” group corresponds to cells transfected with miR‐33 mimic and miR Inhibitor control and “miR‐33i” group corresponds to cells transfected with miR‐33 mimic and miR‐33 Inhibitor. Proteins were isolated with RIPA solution.

#### 2.13.1 Phagocytosis assays

The Beta‐Amyloid (1‐42) Aggregation Kit (rPeptide; A‐1170‐02) was used to prepare Aβ aggregates. We then tagged our Aβ aggregates with pHrodo Red (Thermo Fisher Scientific; P36600) following the iCell Microglia Application protocol (FujiFilm Cellular Dynamics). pHrodo labelled zymosan particles (Thermo Fisher Scientific; P35364) were prepared according to the manufacturer's instructions. After transfection, the media was removed, and BV2 cells were treated with either 100 nM pHrodo‐Aβ aggregates in Opti‐MEM media for 8 hours or with 50 µg/mL of pHrodo labelled zymosan particles in Opti‐MEM media for 8 hours. Differences in uptake were determined by measuring the 560/585 nm excitation/emission signal using the Agilent Synergy H1 Hybrid Multi‐Mode Plate Reader. We determined cell cytotoxicity with the CyQUANT LDH Cytotoxicity Assay (Invitrogen; C20300). We determined changes in cell viability by a colorimetric MTT assay. Representative images were taken on the Leica DMi8 inverted microscope at 20x magnification.[Fig alz14243-fig-0001], [Fig alz14243-fig-0002]

#### 2.13.2 Scratch‐wound assay

BV2 cells plated on a 96‐well plate were scratched with the Incucyte 96‐Well Woundmaker Tool. Plates were placed in the OKO Lab UNO‐T microscope incubator and placed in the Leica Thunder DMi8 microscope. Brightfield images at 5x magnification were taken at time 0 and 24 hours. To quantify wound healing, we used the ImageJ plugin “Wound‐healing‐size‐tool.”^38^ The size of the wound at time 0 hours is the initial wound area, and the wound coverage area relative to the initial wound area is quantified as a percentage at time 24 hours:

$$(1-\left[\mathrm{Wound}\;\mathrm{Area}\;T_{24}/\mathrm{Wound}\;{\mathrm{AreaT}}_{0}\right])\times 100$$

### 2.14 Experimental design and statistical analyses

Sacrifice, tissue collection, and data collection were performed blind to the investigator. All bar graphs show the mean ± standard error of the mean (SEM). Statistical tests were performed in GraphPad Prism 8 software. Statistical analyses were performed by using an unpaired two‐tailed *t* test.

## 3 RESULTS

### 3.1 Deletion of *mir‐33* reduces the levels of insoluble Aβ peptides and plaque deposition

**FIGURE 1 alz14243-fig-0001:**
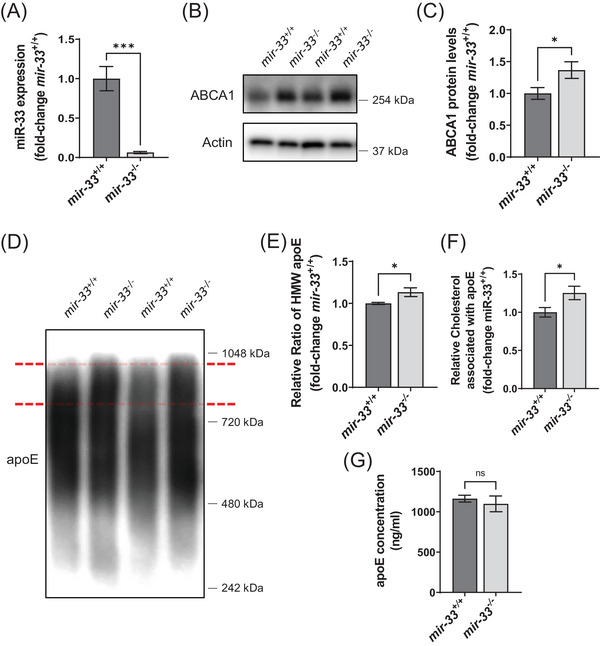
Deletion of *mir‐33* increases ABCA1 protein levels and increases the lipidation of apoE in *APP/PS1* mice. A, miR‐33 expression was determined by qPCR from RNA isolated from cortical tissue of *mir‐33* ^+/+^;*APP/PS1* and *mir‐33* ^−/−^;*APP/PS1* mice. Representative western blot (B) and quantification (C) of ABCA1 protein levels from hippocampal RIPA lysate (normalized to β‐actin protein levels). D, Representative western blots probed for apoE in its native condition run without detergents. The red lines indicate the HMW apoE species. E, Quantification of the HMW apoE bands relative to the total apoE signal for each sample. F, Quantification of the cholesterol associated with apoE that was immunoprecipitated from cortical PBS lysate. G, Quantification of apoE protein levels from hippocampal PBS lysate determined by ELISA. All values are mean ± SEM. ***p < 0.001 | *p < 0.05 | NS, not significant (unpaired two‐tailed *t* test; *n* = 5–6 for *mir‐33* ^+/+^;*APP/PS1*, *n* = 5 for *mir‐33* ^−/−^;*APP/PS1*). apoE, apolipoprotein E; ELISA, enzyme‐linked immunosorbent assay; HMW, high molecular weight; PBS, phosphate‐buffered saline; qPCR, quantitative polymerase chain reaction; RIPA, radioimmunoprecipitation assay; SEM, standard error of the mean.

To validate our mouse model, we measured miR‐33 expression in the brains of *mir‐33* ^−/−^ mice. miR‐33 levels in the cortex were reduced by 94% compared to *mir‐33* ^+/+^ mice (t[9] = 5.498, *p* = 0.0004, unpaired two‐tailed *t* test; Figure 1A). We also found that deletion of *mir‐33* significantly increased ABCA1 protein levels (t[9] = 2.372, *p* = 0.0418, unpaired two‐tailed *t* test; Figure 1B‐C) and increased the level of high molecular weight (HMW) apoE (t[8] = 2.529, *p* = 0.0353, unpaired two‐tailed *t* test), suggesting higher lipidation of apoE (Figure 1D‐E). To directly test whether the lipidation of apoE was increased by *mir‐33* gene deletion, we immunoprecipitated apoE from cortical PBS lysate and measured the levels of cholesterol associated with apoE (Figure 1F). We found that the deletion of *mir‐33* significantly increased the lipidation of apoE (t[9] = 2.423, *p* = 0.0384). To determine whether the level of apoE protein was also affected by *mir‐33* deletion, we performed an apoE ELISA experiment (Figure 1G). There was no difference in the levels of apoE between our genotypes (t[9] = 0.645, *p* = 0.5353). This finding is consistent with a previous report that demonstrated no change in apoE protein levels when ABCA1 was overexpressed 2‐ or 6‐fold over the endogenous level in the cortex.^13^

**FIGURE 2 alz14243-fig-0002:**
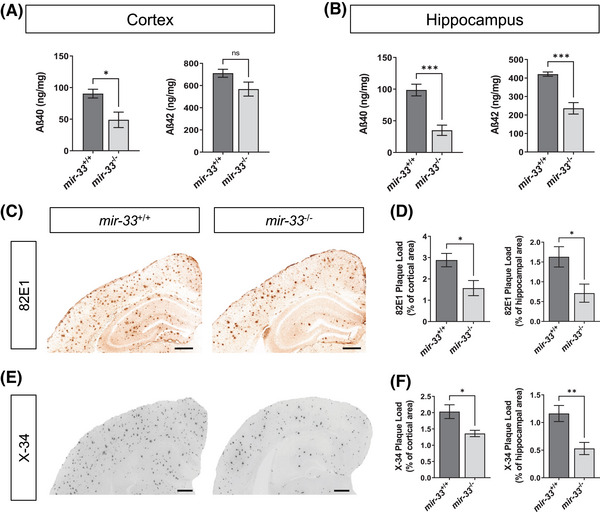
Deletion of *mir‐33* decreases insoluble Aβ peptides and Aβ plaque deposition in *APP/PS1* mice. Aβ levels were measured using the MSD Aβ ELISA kit. Insoluble Aβ_40_ and Aβ_42_ levels were measured from cortical (A) and hippocampal (B) guanidine fractions. C, Representative images of brain sections immunostained with Aβ‐specific 82E1 antibody. D, Quantification of plaque load in cortical and hippocampal areas. E, Representative images of brain sections stained with X‐34 dye that detects fibrillar plaques. F, Quantification of X34+ fibrillar plaque load in cortical and hippocampal areas. All values are mean ± SEM. Scale bars equal to 500 µm. *p < 0.05 | **p < 0.01 | ***p < 0.001 (unpaired, two‐tailed *t* test; *n* = 6 for *mir‐33* ^+/+^;*APP/PS1*, *n* = 5 for *mir‐33* ^−/−^;*APP/PS1*). Aβ, amyloid beta; ELISA, enzyme‐linked immunosorbent assay; MSD, Meso Scale Discovery; SEM, standard error of the mean.

To determine whether loss of *mir‐33* affects the levels of insoluble Aβ peptides in our *APP/PS1* mouse model, we quantified the levels of Aβ_40_ and Aβ_42_ peptides in the guanidine fractions of the cortices and hippocampi of *mir‐33* ^+/+^;*APP/PS1* and *mir‐33* ^−/−^;*APP/PS1* mice using the MSD quantitative immunoassay. Loss of *mir‐33* significantly reduced Aβ_40_ peptide levels by 46% and 64% in the cortex (t[9] = 3.096, *p* = 0.0128, unpaired two‐tailed *t* test) and hippocampus (t[9] = 5.035, *p* = 0.0007, unpaired two‐tailed *t* test), respectively (Figure 2A‐B). There was also a significant reduction of Aβ_42_ peptide levels in the hippocampus by 44% (t[9] = 5.983, *p* = 0.0002, unpaired two‐tailed *t* test; Figure 2B). Although there was a trend toward Aβ_42_ peptide reduction in the cortex, it did not reach statistical significance (t[9] = 2.057, *p* = 0.0698, unpaired two‐tailed *t* test; Figure 2A).

Given the dramatic decrease in Aβ peptide levels we observed, we next determined whether the deletion of *mir‐33* in the *APP/PS1* mouse model reduced the deposition of amyloid plaques. We immunostained brain sections with an anti‐Aβ antibody (82E1; Figure 2C), which specifically recognizes the N‐terminal end of human Aβ peptide. Consistent with our biochemical Aβ peptide quantification, we found that deletion of *mir‐33* significantly reduced Aβ plaque deposition by 46% and 56% in the cortex (t[9] = 2.796, *p* = 0.0208, unpaired two‐tailed *t* test) and hippocampus (t[9] = 2.610, *p* = 0.0283, unpaired two‐tailed *t* test), respectively (Figure 2D). In addition to 82E1 immunohistochemistry, we stained brain sections with the X‐34 fluorescent stain (Figure 2E). While the 82E1 antibody will recognize all Aβ peptide‐positive plaques, X‐34 recognizes β‐sheet structures present in amyloid plaques.^39^ We found that loss of *mir‐33* significantly reduces X‐34–positive cored plaque load by 33% in the cortex (t[9] = 2.699, *p* = 0.0244, unpaired two‐tailed *t* test) and 54% in the hippocampus (t[9] = 3.350, *p* = 0.0085, unpaired two‐tailed *t* test), respectively (Figure 2F). Taken together, our quantitative and immunohistochemical analyses demonstrated that the deletion of *mir‐33* reduces amyloid pathology.

### 3.2 Deletion of *mir‐33* does not alter the levels of proteins regulating the production, degradation, or clearance of Aβ

To investigate a possible mechanism by which *mir‐33* regulates Aβ accumulation (Figure 2), we first determined whether the levels of proteins involved in the production or degradation of Aβ were altered by the loss of *mir‐33* gene. The amyloidogenic pathway consists of the initial cleavage of full‐length APP by β‐secretase (BACE1) and subsequent cleavage by γ‐secretase (PSEN), which yields Aβ.^1^ In addition to the amyloidogenic pathway, enzymes such as neprilysin (NEP) and insulin‐degrading enzyme (IDE) can counteract this process by degrading Aβ, highlighting the complex regulation of Aβ metabolism.^40^ The deletion of *mir‐33* did not alter protein levels of APP (t[9] = 0.6446, *p* = 0.5353, unpaired two‐tailed *t* test), PSEN1 (t[9] = 0.0592, *p* = 0.9541, unpaired two‐tailed *t* test), NEP (t[9] = 0.4981, *p* = 0.6304, unpaired two‐tailed *t* test), or IDE (t[9] = 0.6291, *p* = 0.5449, unpaired two‐tailed *t* test; Figure S1A in supporting information). The loss of *mir‐33* resulted in a modest but significant decrease in BACE1 protein levels (t[9] = 2.337, *p* = 0.0443, unpaired two‐tailed *t* test; Figure S1A). To determine whether this small decrease in BACE1 protein level had any functional effect, we performed western blot probing for the amyloidogenic cleavage product β‐CTF, produced by the BACE1 enzyme (Figure S1B). The loss of *mir‐33* did not change the levels of β‐CTF (t[9] = 0.0141, *p* = 0.9891, unpaired two‐tailed *t* test; Figure S1C). These findings suggest that the reduction in Aβ peptide levels and plaque deposition was not due to the decreased amyloidogenic APP processing or by the increased degradation of Aβ by enzymes, such as NEP or IDE.

Furthermore, receptors that bind to apoE have been shown to influence amyloid pathology.^21^, ^41^ Because the deletion of *mir‐33* regulates apoE lipidation, we aimed to determine whether the expression of apoE receptors was affected. Loss of *mir‐33* did not affect *Ldlr* mRNA level (t[9] = 1.160, *p* = 0.2758; Figure S2A in supporting information) or LDLR protein levels (t[9] = 0.292, *p* = 0.7772; Figure S2B). Additionally, *mir‐33* deletion also did not affect *Lrp1* mRNA level (t[9] = 0.646, *p* = 0.5347; Figure) or LRP1 protein levels (t[9] = 0.421, *p* = 0.6838; Figure S2D).

### 3.3 Deletion of *mir‐33* reduces astrocyte and microglial activation

**FIGURE 3:**
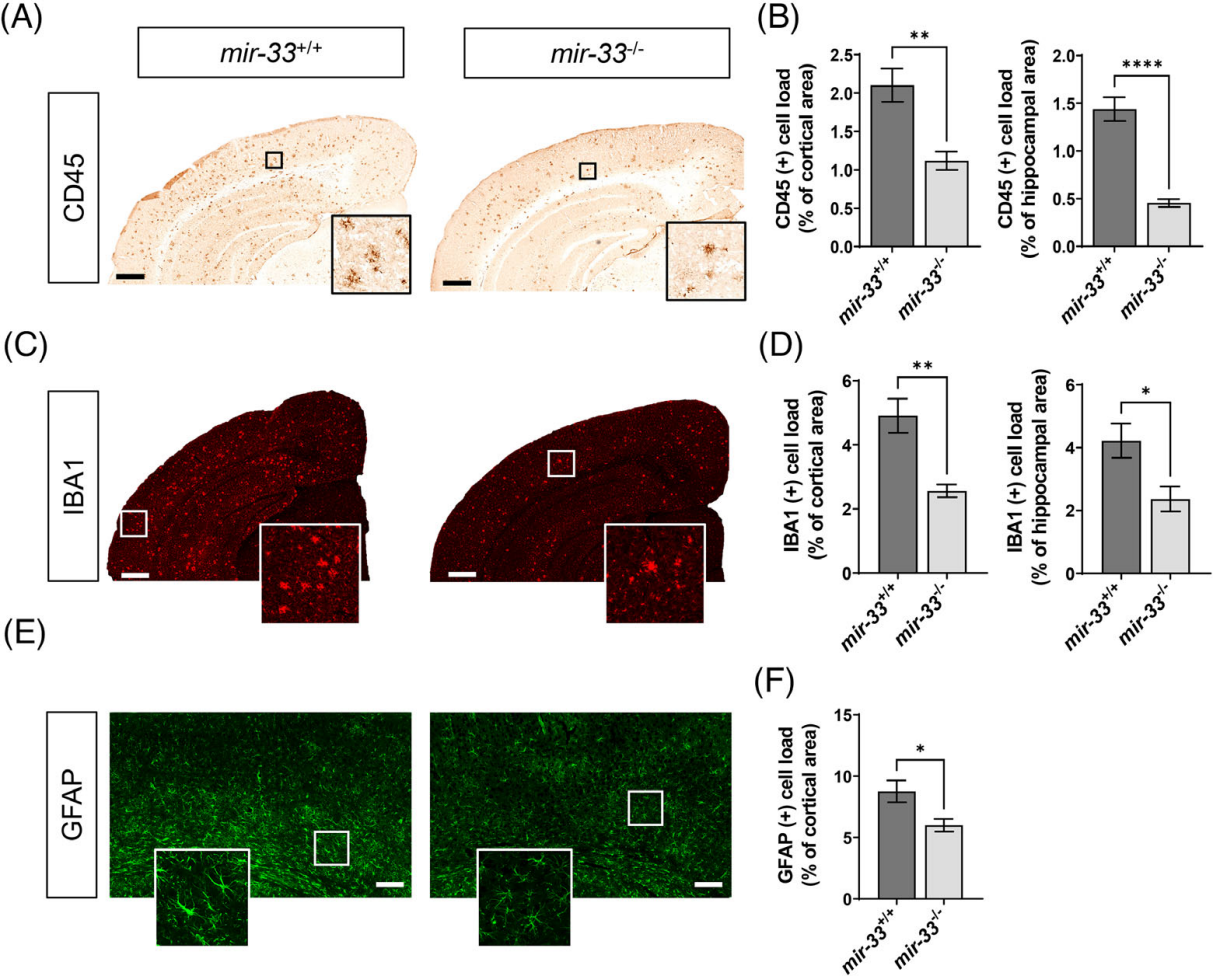
Deletion of *mir‐33* ameliorates gliosis in *APP/PS1* mice. A, Representative images of brain sections immunostained with anti‐CD45 antibody. B, Quantification of CD45+ cell load in cortical and hippocampal areas. C, Representative image of brain sections immunostained with anti‐IBA1 antibody. D, Quantification of IBA1+ cell load in cortical and hippocampal areas. E, Representative image of brain sections immunostained with anti‐GFAP antibody. F, Quantification of cortical GFAP+ cell load. All values are mean ± SEM. Scale bars equal to 500 µm. *p < 0.05 | **p < 0.01 (unpaired two‐tailed *t* test; *n* = 6 for *mir‐33* ^+/+^;*APP/PS1*, *n* = 5 for *mir‐33* ^−/−^;*APP/PS1*). GFAP, glial fibrillary acidic protein; IBA1, ionized calcium binding adaptor molecule 1; SEM, standard error of the mean.

In addition to amyloid pathology, this *APP/PS1* mouse model exhibits aberrant glial activation. Recent studies have demonstrated that the dysfunction of astrocytes and microglia is implicated in the pathogenesis of AD.^42^ Therefore, we determined whether the loss of *mir‐33* would modulate the activation of astrocytes and microglia. To determine whether *mir‐33* deletion affects microglial activation, we stained brain sections with an anti‐CD45 antibody (Figure 3A) and an anti‐IBA1 antibody (Figure 3C). CD45 is expressed on resting and activated microglia as well as most cells from the hematopoietic lineage, and it is increased in the brains of patients with AD.^43^ The loss of *mir‐33* reduced the CD45‐positive cell load by 47% and 68% in the cortex (t[9] = 3.727, *p* = 0.0047, unpaired two‐tailed *t* test) and hippocampus (t[9] = 6.913, *p* = < 0.0001, unpaired two‐tailed *t* test), respectively (Figure 3B). IBA1 is a marker of both resting and activated microglia. Activated microglia have increased IBA1 expression, and this marker is commonly used in immunohistochemistry to determine changes in microglial activation.^44^ Consistent with our CD45 results, the deletion of *mir‐33* reduced the IBA1‐positive cell load by 48% and 44% in the cortex (t[9] = 3.801, *p* = 0.0042, unpaired two‐tailed *t* test) and hippocampus (t[9] = 2.646, *p* = 0.0266, unpaired two‐tailed *t* test), respectively (Figure 3D). Taken together, these data demonstrate that the loss of *mir‐33* reduces microglial activation in this *APP/PS1* mouse model.

To determine changes in the extent of astrocyte activation, we stained brain sections with an anti‐GFAP antibody (Figure 3E). GFAP expression is increased in the brains of AD patients, and it is commonly used to determine changes in the activation of astrocytes.^45^ The loss of *mir‐33* resulted in a 33% reduction in GFAP‐positive cell load in the cortex (t[9] = 2.555, *p* = 0.0309, unpaired two‐tailed *t* test; Figure 3F). We did not quantify the extent of GFAP staining in the hippocampus because the basal level of GFAP signals was too high for reliable quantification. Overall, the deletion of *mir‐33* significantly reduced glial activation in this *APP/PS1* mouse model.

### 3.4 Multi‐omics analyses indicate that miR‐33 regulates microglial activation and microglial migration

**FIGURE 4:**
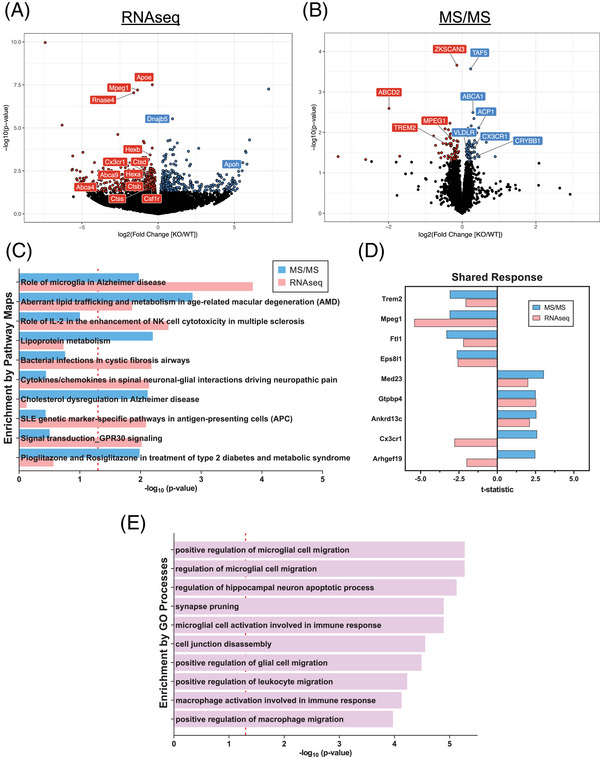
Multi‐omics analyses suggest that miR‐33 may regulate microglial migration and immune response. A, Volcano plot visualizing the 882 DEGs identified by bulk RNA‐sequencing (RNAseq) from cortical tissue between *mir‐33* ^+/+^;*APP/PS1* (WT) and *mir‐33* ^−/−^;*APP/PS1* (KO) mice. B, Volcano plot visualizing the 125 DAPs identified by mass spectrometry (MS/MS) from cortical lysate between *mir‐33* ^+/+^;*APP/PS1* (WT) and *mir‐33* ^−/−^;*APP/PS1* (KO) mice. C, Pathway Map enrichment analysis was performed with the DEGs (red) and DAPs (blue) identified between *mir‐33* ^+/+^;*APP/PS1* and *mir‐33* ^−/−^;*APP/PS1* mice using the MetaCore software. The top 10 Pathway Maps are shown with the vertical red line denoting significant Pathway Maps identified. D, The shared response are the common DEGs and DAPs between RNAseq and MS/MS visualized as a function of their *t* statistic. E, GO enrichment analysis was performed with the shared response common DEGs and DAPs using the MetaCore software. The top 10 GO Processes are shown with the vertical red line denoting significant GO Processes identified. DAPs, differentially abundant proteins; DEGs, differentially expressed genes; GO, Gene Ontology; KO, knock out; WT, wild type.

We demonstrated the deletion of *mir‐33* increases ABCA1 protein levels and apoE lipidation (Figure 1). However, given the nature of miR regulation, in which a single miR can be predicted to regulate the expression of multiple genes, we aimed to understand the global transcriptomic and proteomic changes between *mir‐33* ^+/+^;*APP/PS1* and *mir‐33* ^−/−^;*APP/PS1* mice. We performed bulk RNA‐seq and TMT‐based mass spectrometry to identify the differentially expressed genes (DEGs) and the differentially abundant proteins (DAPs) in the cortex. We identified 882 DEGs (Figure 4A) and 128 DAPs (Figure 4B). We performed integrated pathway analyses using MetaCore to gain insight into the pathway changes caused by the deletion of *mir‐33* in the context of amyloid pathology. We identified the top 10 Pathway Maps that were significantly enriched in our two datasets using Pathway Map enrichment in MetaCore (Figure 4C). Interestingly, the two Pathway Maps that were significantly regulated in both datasets were the “Role of microglia in Alzheimer's disease” and the “Aberrant lipid trafficking and metabolism in age‐related macular degeneration” (Table S2 in supporting information). The second pathway, “Aberrant lipid trafficking and metabolism in age‐related macular degeneration” increases the validity of our Pathway Map approach because this pathway is already known to be regulated by ABCA1, the direct downstream target of miR‐33.

We next determined the shared response of DEGs and DAPs that were common between the RNA‐seq and proteomics datasets (Figure 4D). To identify the pathways and processes regulated by these shared response genes and proteins, we performed GO enrichment analysis using MetaCore (Figure 4E). The GO terms including “positive regulation of microglial cell migration” and “microglial cell activation involved in immune response” were among the top identified GO terms. Notably, CX3CR1 and TREM2 were shared terms that were in both of these GO Processes (Table S3 in supporting information). To determine whether any of the differentially abundant proteins were predicted or validated targets of miR‐33, we performed miR target analysis using an R package “multiMir.” *Cx3cr1* and *Trem2* are not predicted targets of *mir‐33* (Table S4 in supporting information), suggesting the change in their levels is likely through the secondary downstream effect of *mir‐33* deletion.

### 3.5 Inhibiting miR‐33 function facilitates microglial migration and Aβ phagocytosis

**FIGURE 5:**
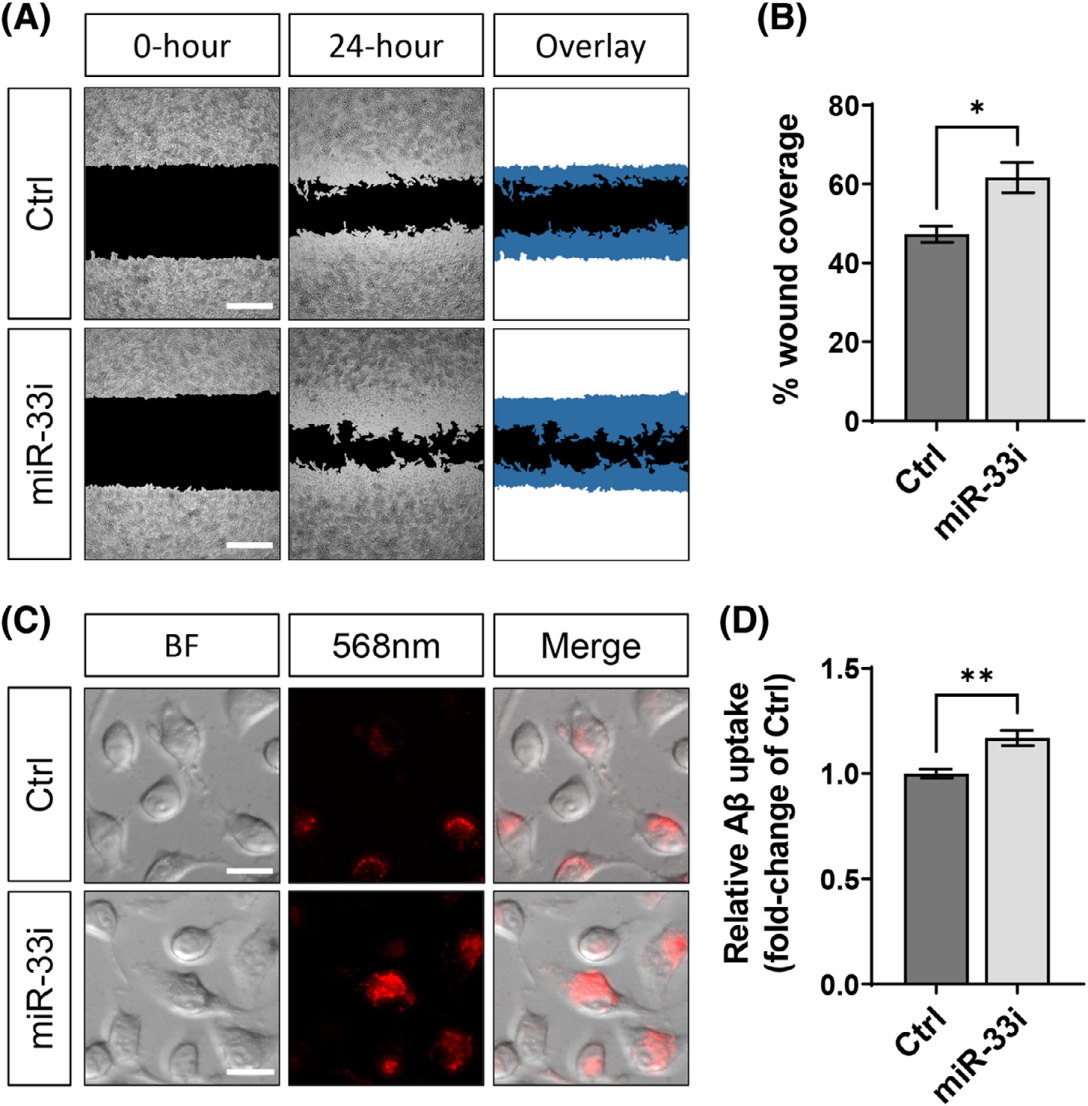
Inhibition of miR‐33 increases microglial migration and Aβ phagocytosis. A, Scratch‐wound assay was performed with BV2 cells to compare between the control (Ctrl) group and the miR‐33 inhibitor group. Twenty‐four hours after transfection, a wound was created and imaged at 0 hours, and 24 hours. The overlay demonstrates the remaining wound area (black) and the area migrated by the BV2 cells (blue). B, Quantification of the wound coverage area by BV2 cells at time 24 hours. C, Aβ phagocytosis assay performed with transfected BV2 cells comparing the Ctrl versus the miR‐33 inhibitor group. Twenty‐four hours after transfection, cells were treated with Aβ aggregates tagged with pHrodo and the 560/585 nm fluorescent signal was measured after 8 hours. D, Quantification of the relative change in pHrodo fluorescent signal compared to Ctrl. All values are mean ± SEM. Scale bars equal to 200 µm (A) and 10 µm (C). *p < 0.05 | **p < 0.01 (unpaired two‐tailed *t* test; *n* = 5 for scratch‐wound assay, *n* = 6 for Aβ uptake assay). Aβ, amyloid beta; SEM, standard error of the mean.

Given that our multi‐omics analyses implicate both microglial migration and its activation (Figure 4), we aimed to functionally validate the role of these GO processes using mechanistic cell biology approaches. In AD brains, microglia migrate toward amyloid plaques and phagocytose Aβ, contributing to the clearance of Aβ.^46^ To test the hypothesis that the loss of *mir‐33* increases microglial migration, we inhibited miR‐33 in microglial cells and performed a scratch‐wound assay (Figure 5A). Transfection with our constructs did not affect cell viability (t[4] = 1.020, *p* = 0.3652, unpaired two‐tailed *t* test; Figure S3A in supporting information). Inhibiting miR‐33 in microglia increased wound coverage by 27% (t[8] = 3.282, *p* = 0.0112, unpaired two‐tailed *t* test) at 24 hours post scratch (Figure 5B).

To determine the potential role of miR‐33 in regulating Aβ phagocytosis by microglia, we inhibited miR‐33 in microglial cells and treated them with pHrodo‐tagged Aβ aggregates (Figure 5C). Treatment of Aβ aggregates did not induce cell cytotoxicity (t[6] = 0.5235, *p* = 0.6194, unpaired two‐tailed *t* test; Figure S3B). Treatment with a miR‐33 inhibitor increased Aβ uptake by 17% (t[10] = 4.030, *p* = 0.0024, unpaired two‐tailed *t* test; Figure 5D). Importantly, ABCA1 protein levels were increased by 54% when we inhibited miR‐33 activity in these microglia (t[10] = 3.643, *p* = 0.0045, unpaired two‐tailed *t* test; Figure S3C‐D), confirming the functional effect of the miR‐33 inhibitor on its validated target gene, *Abca1*. Additionally, we determined whether the increased microglial phagocytosis through inhibition of miR‐33 would increase the uptake of another ligand. Therefore, we determined whether miR‐33 also regulates the uptake of zymosan particles that have been commonly used to assess microglial function.^25^ We inhibited miR‐33 activity in microglia with anti‐miR‐33 oligonucleotide and treated cells with pHrodo‐labeled zymosan particles (Figure S4A in supporting information). Inhibition of miR‐33 activity increased the uptake of pHrodo‐labeled zymosan particles by 1.91 fold (t[10] = 7.662, *p* < 0.0001; Figure S4B). Taken together, we found that miR‐33 inhibition increases the migration of microglia and increases their phagocytic function.

## 4 DISCUSSION

miRs have emerged as a new class of small, inhibitory molecules capable of inhibiting the expression of their target genes. Previously, miR‐33 was shown to negatively regulate ABCA1 protein levels.^17^, ^18^ Although the significance of ABCA1 overexpression in amyloid pathology has been demonstrated in a mouse model,^13^ there was a lack of human data linking *ABCA1* to an increased risk of developing AD. Recently, however, the *ABCA1* gene was identified as a novel genetic risk factor for AD.^5^, ^6^ Therefore, miR‐33 is a potential target for modulating ABCA1 protein levels and ameliorating amyloid pathology. In this study, we demonstrate for the first time that the deletion of *mir‐33* in an amyloidosis mouse model significantly increases ABCA1 protein levels, increases apoE lipidation, and reduces the levels of insoluble Aβ peptides and amyloid plaque deposition. We also showed that the activation of glial cells in the brain was significantly reduced in our *mir‐33* ^−/−^;*APP/PS1* mice.

Lipidation of apoE has recently emerged as a promising therapeutic target for AD.^47^ In humans, three major isoforms of *APOE* are lipidated in an isoform‐specific manner (ε2 > ε3 > ε4).^9^, ^48^ Interestingly, this lipidation pattern inversely correlates with the risk of developing AD (ε2 < ε3 < ε4). Moreover, patients with mild cognitive impairment or AD exhibit a 30% lower ABCA1‐mediated cholesterol efflux capacity compared to cognitively healthy participants.^49^ This suggests that not only is cholesterol efflux altered by *APOE* isoforms, but also by ABCA1 function in the disease state. This further highlights the importance of finding modulators to activate this ABCA1–APOE lipidation pathway. The process by which ABCA1 lipidates apoE is mediated mainly by astrocytes. Indeed, both *ABCA1* and *APOE* are highly expressed in astrocytes in the brain.^50^, ^51^ Interestingly, some *mir* genes are located within the introns of host genes and can be co‐expressed with their host genes. *Mir‐33*, which is encoded by intron 16 of the *Srebf2* gene, is coexpressed with *Srebf2*.^15^ Therefore, *Srebf2* expression can be used as a proxy to gain some insight into miR‐33 levels in different cell types. In line with *ABCA1* and *APOE* expression, *SREBF2* is most highly expressed in astrocytes in the brain.^50^, ^51^ We therefore initially hypothesized that any changes in amyloid pathology could be driven by the role of miR‐33 in astrocytes. However, it is important to note that other cell types, notably microglia, also can express relatively high levels of both *ABCA1* and *APOE*, especially under certain conditions. Indeed, *APOE* expression in microglia is increased in the brains of AD patients and is a marker for disease‐associated microglia (DAM).^52^, ^53^ Furthermore, *ABCA1* deficiency has been shown to increase inflammatory response and lipid accumulation in microglia.^54^, ^55^ Therefore, it is important to consider that microglial lipid metabolism may also be contributing to the observed reduction in amyloid pathology in these mice.

Our multi‐omics analyses revealed Pathway Maps and GO terms associated with the microglial immune response and microglial migration. Microglia constantly survey and move within the central nervous system and respond to pathogens, injuries, and other irregularities. Specifically, in AD, microglia are integral for the clearance of Aβ plaques.^56^ In our mouse model, *mir‐33* is knocked out in all cell types; therefore, these enrichment terms cannot be attributed to the loss of *mir‐33* in any specific cell type. Regarding Aβ plaques, apoE is integral to plaque dynamics.^57^, ^58^ ApoE binds Aβ and is found in abundance within Aβ plaques. This apoE–Aβ interaction within plaques modulates how microglia respond to plaques. For example, apoE binds to Aβ and facilitates its clearance by microglia through the binding of TREM2.^59^ Importantly, the extent of apoE lipidation can inhibit this binding.^59^ In addition, the apoE found within Aβ plaques is non‐lipidated or poorly lipidated.^60^ Therefore, it is possible that the enrichment terms associated with the microglial immune response and microglial migration might be a secondary response to the highly lipidated apoE secreted primarily by astrocytes in *mir‐33* knockout mice. However, the loss of *mir‐33* specifically within microglia could also drive these changes in the microglial immune response and microglial migration. Therefore, we used in vitro assays to determine whether inhibition of miR‐33 in microglia alone modulates their migration and the uptake of Aβ. Our study demonstrated that inhibiting miR‐33 significantly increased microglial migration and increased the uptake of Aβ aggregates and zymosan particles. These findings represent the first functional evidence implicating miR‐33 in regulating microglial migration and phagocytosis.

Recent research highlights the critical role of these microglial functions in regulating amyloid pathology.^25^, ^61^ Karahan et al. demonstrated that genetically deleting Abi3, a protein essential for cytoskeletal dynamics, significantly impaired the migration and phagocytosis of microglia.^25^ This leads to significant increases in the levels of insoluble Aβ peptides and amyloid plaque deposition in the brains of 5XFAD mice. Interestingly, in our study, the deletion of *mir‐33* increased the ability of microglia to take up Aβ and to migrate. Further studies are warranted to understand how miR‐33 regulates these functions within microglia. Importantly, it is worth highlighting that some of our DEGs and DAPs might be indirect responses to the reductions in amyloid pathology we observed. At 8 months of age, this *APP/PS1* mouse model exhibits severe amyloid pathology and plaque deposition. While it is possible that the response to amyloid pathology could influence our multi‐omics analyses, this discussion underscores the importance of functionally validating enrichment results to confirm their validity, as demonstrated in this study (Figure 5).

While we confirmed that inhibition of miR‐33 increases Aβ uptake and microglial migration, our current study cannot determine whether the changes we observed in vivo are primarily driven by this mechanism, or by the astrocyte‐mediated ABCA1–APOE lipidation mechanism, or if both are necessary for reducing amyloid pathology. Additionally, miRs often display cell type–specific regulation of their targets.^62^, ^63^ We identified potential cell‐type–specific pleiotropic effects that miR‐33 regulates in astrocytes and microglia. The increased lipidation of apoE from astrocytes could enhance the clearance of Aβ by microglia. Moreover, the inhibition of miR‐33 within microglia increases both their ability to migrate and their capacity to phagocytose Aβ. This coordinated effort of *mir‐33* deletion through distinct mechanisms across cell types indicates that miR‐33 is a potential therapeutic target for reducing amyloid pathology.

In this study, we demonstrated that the deletion of *mir‐33* significantly reduces amyloid pathology in an *APP/PS1* mouse model. Importantly, our mechanistic experiments revealed that the inhibition of miR‐33 increases the microglial uptake of Aβ and increases microglial migration. Future studies are warranted to identify the mechanism(s) by which miR‐33 regulates Aβ phagocytosis by microglia and their migration. It will be interesting to determine whether such mechanism(s) are dependent on ABCA1 or other targets of miR‐33. In summary, we identified a potential therapeutic target for ameliorating several aspects of amyloid pathology in AD.

## CONFLICT OF INTEREST STATEMENT

The authors declare no conflicts of interest. Author disclosures are available in the supporting information.

## CONSENT STATEMENT

No human subjects participated in this study; consent was not necessary.

## Supporting information

Supporting Information

Supporting Information

Supporting Information

Supporting Information

Supporting Information

Supporting Information
