# Supplementary material for: Deletion of miR‐33, a regulator of the ABCA1–APOE pathway, ameliorates neuropathological phenotypes in APP/PS1 mice
Source: Alzheimers Dement. 2024 Sep 30;20(11):7805–18. doi: 10.1002/alz.14243 (PMC11567857; doi:10.1002/alz.14243)
Supplement: Supplementary file 3 — Supporting Information [file ALZ-20-7805-s001.pdf]

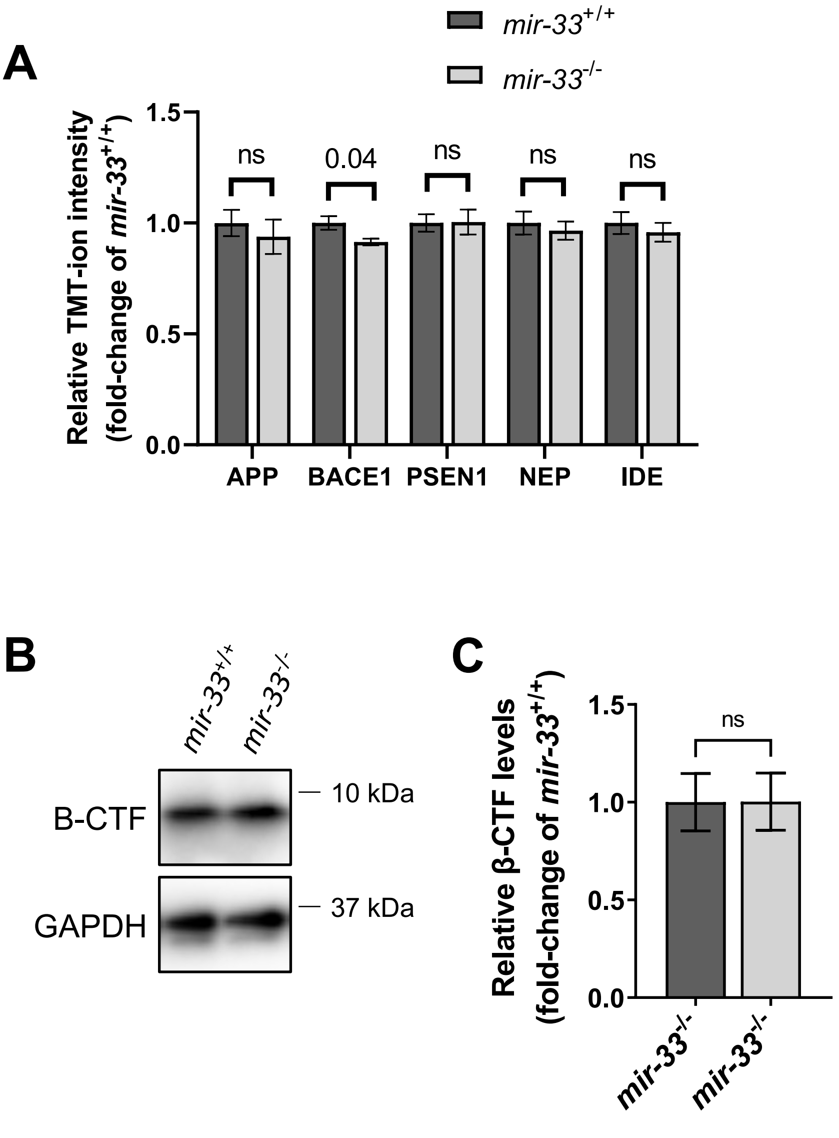
A
mir-33+/+
mir-33-/-
Relative TMT-ion intensity (fold-change of mir-33+/+)
1.5
1.0
0.5
0.0
ns
0.04
ns
ns
ns
APP
BACE1
PSEN1
NEP
IDE
B
mir-33+/+
mir-33-/-
B-CTF
GAPDH
10 kDa
37 kDa
C
Relative β-CTF levels (fold-change of mir-33+/+)
1.5
1.0
0.5
0.0
ns
mir-33-/-
mir-33-/-

Supplemental Figure 1 | The levels of proteins involved in Aβ production and degradation are unchanged between *mir-33*$^{+/+}$;*APP/PS1* and *mir-33*$^{-/-}$;*APP/PS1* mice. (**A**) Protein levels were determined by the relative TMT-ion intensity from mass spectrometry. The levels of proteins integral for amyloidogenic pathways (APP, BACE1, and PSEN1) are quantified as a relative change compared to *mir-33*$^{+/+}$;*APP/PS1* mice. Proteins responsible for the degradation of Aβ (Neprilysin [NEP] and Insulin-degrading enzyme [IDE]) are quantified as a relative change compared to *mir-33*$^{+/+}$;*APP/PS1* mice. (**B**) Representative western blot probed with anti-82E1 antibody recognizing β-CTF fragment of Aβ from cortical RIPA-soluble fractions. (**C**) Quantification of the relative β-CTF levels (normalized to GAPDH protein levels) determined by the 82E1 western blot in (B). All values are mean ± SEM. NS: not significant ([**A** and **C**] unpaired two-tailed t-test; n=6 for *mir-33*$^{+/+}$;*APP/PS1*, n=5 for *mir-33*$^{-/-}$;*APP/PS1*).
