# Supplementary material for: Deletion of miR‐33, a regulator of the ABCA1–APOE pathway, ameliorates neuropathological phenotypes in APP/PS1 mice
Source: Alzheimers Dement. 2024 Sep 30;20(11):7805–18. doi: 10.1002/alz.14243 (PMC11567857; doi:10.1002/alz.14243)
Supplement: Supplementary file 4 — Supporting Information [file ALZ-20-7805-s004.pdf]

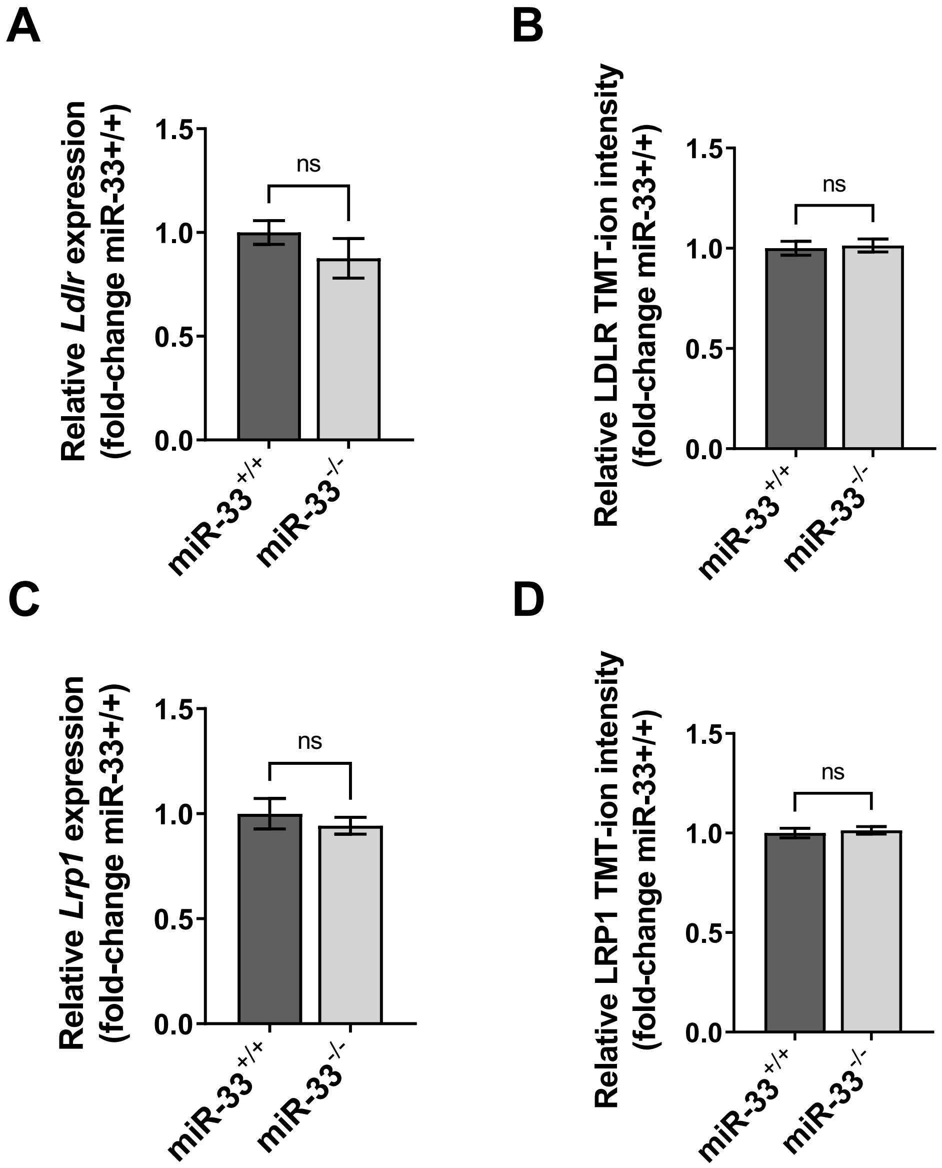
A
Relative Ldlr expression
(fold-change miR-33+/+)
1.5
1.0
0.5
0.0
ns
miR-33+/+
miR-33-/-
B
Relative LDLR TMT-ion intensity
(fold-change miR-33+/+)
1.5
1.0
0.5
0.0
ns
miR-33+/+
miR-33-/-
C
Relative Lrp1 expression
(fold-change miR-33+/+)
1.5
1.0
0.5
0.0
ns
miR-33+/+
miR-33-/-
D
Relative LRP1 TMT-ion intensity
(fold-change miR-33+/+)
1.5
1.0
0.5
0.0
ns
miR-33+/+
miR-33-/-

Supplemental Figure 2 | Levels of LDLR and LRP1 mRNA and protein are unchanged between *mir-33*$^{+/+}$;*APP/PS1* and *mir-33*$^{-/-}$;*APP/PS1* mice. (**A**) Relative levels of *Ldlr* mRNA determined by qPCR. (**B**) Relative LDLR protein levels determined based on the TMT-ion intensity from mass spectrometry data. (**C**) Relative levels of *Lrp1* mRNA determined by qPCR. (**D**) Relative LRP1 protein levels determined based on the TMT-ion intensity from mass spectrometry data. All values are mean ± SEM. NS: not significant (unpaired two-tailed t-test; n=6 for *mir-33*$^{+/+}$;*APP/PS1*, n=5 for *mir-33*$^{-/-}$;*APP/PS1*).
