# Supplementary material for: Deletion of miR‐33, a regulator of the ABCA1–APOE pathway, ameliorates neuropathological phenotypes in APP/PS1 mice
Source: Alzheimers Dement. 2024 Sep 30;20(11):7805–18. doi: 10.1002/alz.14243 (PMC11567857; doi:10.1002/alz.14243)
Supplement: Supplementary file 5 — Supporting Information [file ALZ-20-7805-s005.pdf]

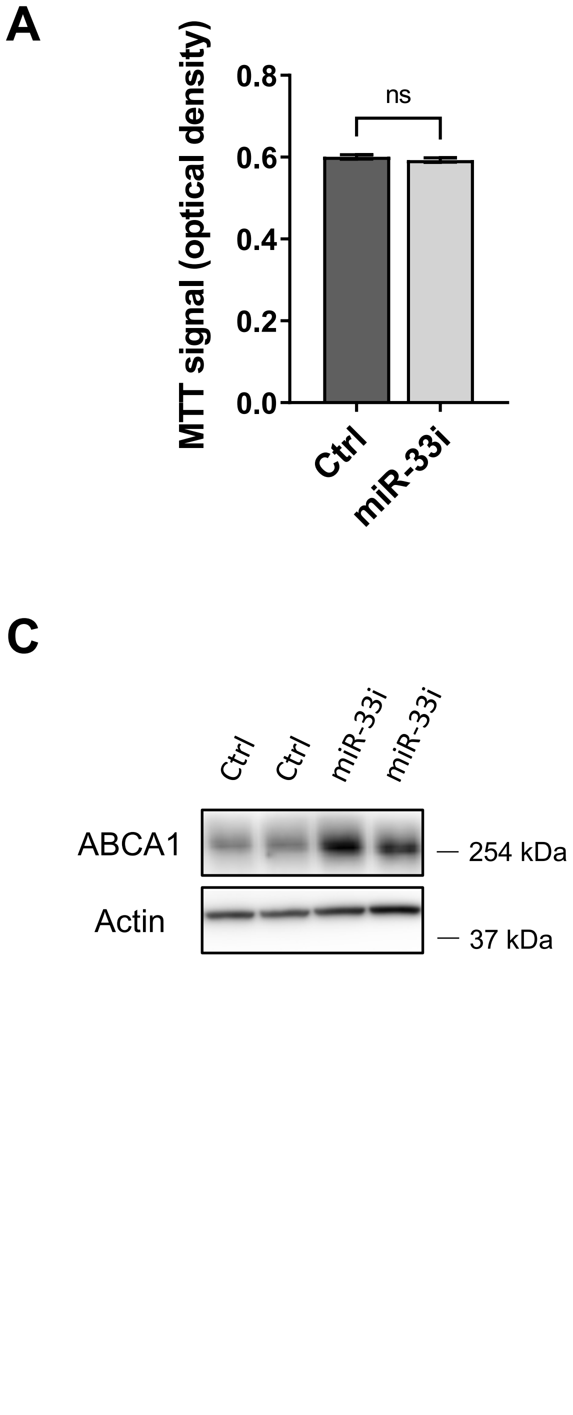

A
MTT signal (optical density)
0.0
0.2
0.4
0.6
0.8
ns
Ctrl
miR-33i
B
Relative LDH release
(fold-change of ctrl)
0.0
0.5
1.0
1.5
ns
Vehicle Ctrl
100nM Aβ
C
Ctrl
Ctrl
miR-33i
miR-33i
ABCA1
Actin
254 kDa
37 kDa
D
ABCA1 protein levels
(fold-change Ctrl)
0.0
0.5
1.0
1.5
2.0
**
Ctrl
miR-33i

Supplemental Figure 3 | Aβ aggregates and inhibition of miR-33 do not induce cell cytotoxicity. (**A**) BV2 microglial cells were treated with 100 nM Aβ aggregates for 24 hours. Relative levels of the LDH released in the media were measured. Vehicle Ctrl is OPTI-MEM media treated BV2 cells (n=4) (**B**) MTT signal from BV2 cells between Ctrl and miR-33i groups 24 hours after transfection (n=3). (**C**) Representative western blot probed with anti-ABCA1 antibody (**D**) Quantification of the relative ABCA1 protein levels between the Ctrl and miR-33i groups (normalized to β-actin protein levels) (n=6). All values are mean ± SEM. **$p<0.01$ | NS-not significant (unpaired two-tailed t-test).
