# Supplementary material for: Deletion of miR‐33, a regulator of the ABCA1–APOE pathway, ameliorates neuropathological phenotypes in APP/PS1 mice
Source: Alzheimers Dement. 2024 Sep 30;20(11):7805–18. doi: 10.1002/alz.14243 (PMC11567857; doi:10.1002/alz.14243)
Supplement: Supplementary file 6 — Supporting Information [file ALZ-20-7805-s003.pdf]

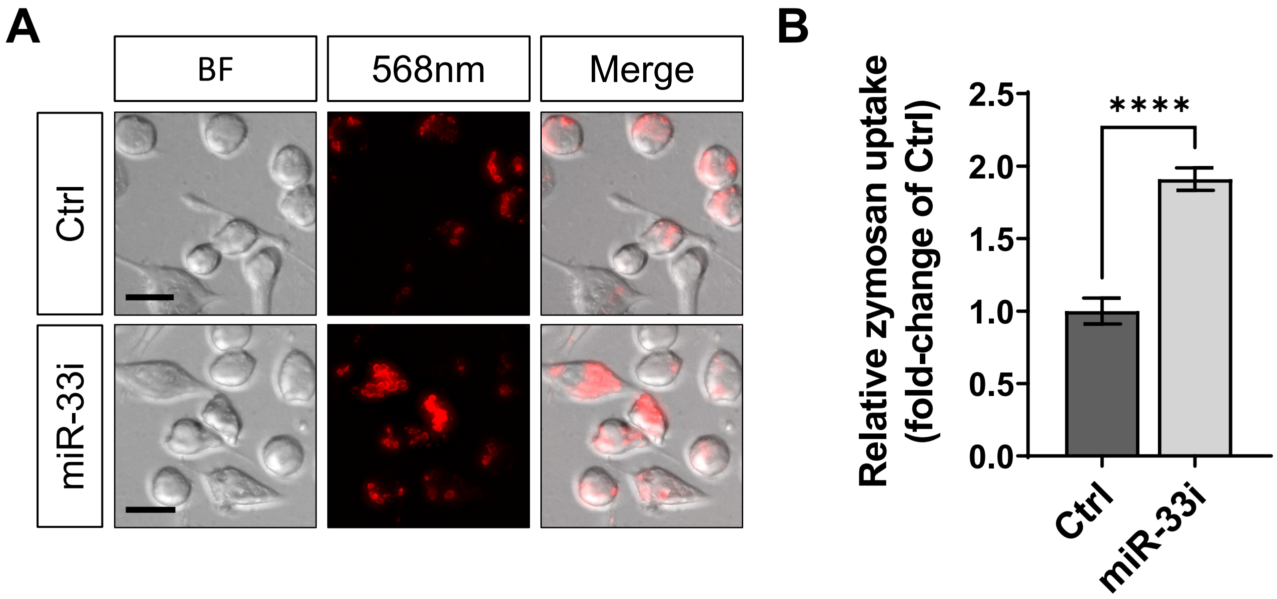
A
BF
568nm
Merge
Ctrl
miR-33i
B
Relative zymosan uptake
(fold-change of Ctrl)
2.5
2.0
1.5
1.0
0.5
0.0
****
Ctrl
miR-33i

Supplemental Figure 4 | Inhibition of miR-33 increases phagocytosis of zymosan particles (**A**) Phagocytosis assay performed with transfected BV2 cells comparing the Ctrl vs the miR-33 inhibitor group. 24 hours after transfection, cells were treated with pHrodo labelled zymosan particles and the 560/585nm fluorescent signal was measured after 8 hours. (**B**) Quantification of the relative change in pHrodo fluorescent signal compared to Ctrl. All values are mean ± SEM. Scale bars equal to 10 µm. *p<0.05 | **p<0.01 (unpaired two-tailed t-test; n=5 for scratch-wound assay, n=6 for Aβ uptake assay). ****p<0.0001 (unpaired two-tailed t-test; n=6).
